# Evaluation of 25% Poloxamer As a Slow Release Carrier for Morphine in a Rat Model

**DOI:** 10.3389/fvets.2018.00019

**Published:** 2018-03-09

**Authors:** Nurul H. Sulimai, Jeff C. Ko, Yava L. Jones-Hall, Hsin-Yi Weng, Meng Deng, Gert J. Breur, Gregory T. Knipp

**Affiliations:** ^1^Department of Veterinary Clinical Sciences, College of Veterinary Medicine, Purdue University, West Lafayette, IN, United States; ^2^Department of Comparative Pathobiology, College of Veterinary Medicine, Purdue University, West Lafayette, IN, United States; ^3^Department of Agricultural and Biological Engineering, Colleges of Agriculture and Engineering, Purdue University, West Lafayette, IN, United States; ^4^Department of Industrial and Physical Pharmacy, College of Pharmacy, Purdue University, West Lafayette, IN, United States; ^5^Purdue Translational Pharmacology CTSI Core, Purdue University, West Lafayette, IN, United States

**Keywords:** poloxamer, morphine, slow release, pharmacokinetic, histopathology, rat

## Abstract

The objectives of this study were to evaluate poloxamer as a slow release carrier for morphine (M) and potential tissue irritation after subcutaneous poloxamer–morphine (PM) injection in a rat model. Based on the result of a previous *in vitro* work, 25% poloxamer, with and without morphine, and saline were administered in 14 rats’ flanks. Blood for morphine concentrations was automatically sampled at multiple preprogrammed time points using the Culex™ unit for 48 h. Skin tissues from the injection sites were harvested and evaluated for histopathological changes. Following M or PM administration, it was determined that the half-life (*t*_1/2_) was significantly longer in the PM (5.5 ± 7.2 h) than M (0.7 ± 0.8 h) indicated a slow dissolution of poloxamer with morphine. The *t*_max_ was within 15 min and *C*_max_ was approximately three times higher with M than with PM, reaching 716.8 (±153.7 ng/ml) of plasma morphine concentrations. There was no significant difference in total area under the curve and clearance of M versus PM. Histology inflammatory scores were similar between M, PM, and poloxamer but were significantly higher than saline control. We concluded that 25% poloxamer was capable of increasing the *t*_1/2_ of morphine, without a significant tissue irritation.

## Introduction

A single administration of a slowly released analgesic that provides a sustained plasma concentration (Cp) above the minimum effective concentration is desirable to provide a longer duration of action for pain control. The advantages of such include reduction of frequent drug administration, reduced site of injection irritation, improved patient comfort and compliance. A steady state of plasma concentrations of an analgesic is routinely achieved by either constant rate infusion or *via* a slowly release mechanism in pain management. Constant rate infusion requires an intravenous access and sustained administration with a syringe pump over time after a loading dose. Risk of disconnection and infection of the intravenous site and discomfort associated with CRI with wires and tubings are the main disadvantages. One of the slow release mechanisms for pain management is using a carrier that is capable of releasing analgesic steadily over a long duration. For example, the development of hydrogel polymeric carrier systems where the drug is at least partially entrapped with the gel and elutes in a manner that provides sustained release.

Poloxamer is a biodegradable tri-block copolymer that is made up of polyethylene oxide and polypropylene oxide (1–5). Poloxamer is one of the hydrogel forming biomaterials ideal for slow releasing of drugs (3). Its thermo-reversible hydrogel forming property, existing in a liquid form at room temperature and forming a gel at physiologically relevant temperatures above 30°C, that makes it attractive as an injectable carrier. Poloxamer is inexpensive, easy to prepare, comes in several different molecular weights that control its gel forming properties, and has been shown to be biocompatible in humans and animals (1, 6, 7). Morphine is an effective and economical analgesic. However, morphine has been shown to have a short *t*_1/2_ between 1 and 2 h that necessitates repeated administration (8–11).

In a separate study, we tested 20, 25, and 30% of poloxamer mixture with morphine and its ability to release morphine in an *in vitro* diffusion well environment under various temperatures and pH solutions. We concluded that 25% poloxamer is capable of slowly releasing morphine *in vitro* (12) under physiological environment. The purposes of this study were to confirm that 25% poloxamer was able to increase morphine *t*_1/2_ when administered subcutaneously in rats. Furthermore, we wanted to evaluate whether 25% poloxamer with morphine causing tissue irritation at the injection site (13). We hypothesized that poloxamer was able to extend morphine’s *t*_1/2_ and the subcutaneous (SC) injection of the poloxamer with morphine is safe.

## Materials and Methods

### Animals and Husbandry

This work was conducted at Purdue University’s AAALAC-accredited facility and was approved by Purdue’s IACUC (1412001174). Fourteen male Sprague-Dawley rats (Sure Harlan, Indianapolis), 5 months old and weighed 300–400 g, were used in this experiment. Before the surgery, the rats were acclimated for at least 1 week. On the day of the surgery, the rat was anesthetized briefly for catheterization. Thereafter, the rats were housed in the BASi Raturn™ system (West Lafayette, IN, USA) for the duration of this study. The Raturn™ is a movement responsive caging system that allows the rats to move in the cage freely while being attached to an automated blood sampling tubing system. They were provided with *ad libitum* food and water while housed in the system after recovery from surgery.

### Poloxamer with Morphine Kinetic Study

#### Catheterization for Blood Sampling

A carotid artery was cannulated surgically in each rat under general anesthesia aseptically. Meloxicam was given orally at 1 mg/kg before the surgery. The rat was induced and maintained with isoflurane in oxygen. The dorsal and ventral side of the neck was prepped, and the skin incision was made to dissect the omohyoid muscle. The carotid artery was exposed, and a catheter was cannulated with the catheter tunneled subcutaneously behind the ear and through the skin in between the scapulae. Once the procedure was completed, the rat was recovered and returned to the Raturn™ and connected to the automated sampling system. The carotid catheter was checked and maintained during the entire study period.

#### Study Design and Treatments

For the pharmacokinetic study of morphine, the rats were assigned to either morphine alone (M, *n* = 7) or morphine with 25% poloxamer [poloxamer–morphine (PM, *n* = 7)] randomly. For the assessment of injection site tissue irritation, each rat’s right and left flanks were injected with the same volume of physiological saline, poloxamer, morphine, or morphine with 25% poloxamer, respectively. The summary of the treatments was presented in Table 1. The morphine was dosed at 2 mg/kg. For the PM mixture, 0.1 ml of commercial injectable morphine (10 mg/ml) was mixed with 0.4 ml of poloxamer 32.14% to yield a final concentration of 25% poloxamer. A 1 cm × 1 cm square area on the right and left flanks of the rats were shaved to demarcate the site for SC injection. Immediately after the rat was injected with the respective treatment drugs, the blood sample (time 0) was withdrawn *via* the automated blood sampling system. Two hundred microliters of blood were collected from each rat at each of the following time points: 0, 10, 30, and 45 min and 2, 4, 6, 8, 12, 24, and 48 h following drug treatments.

**Table 1:** Rats were administered either morphine or morphine with 25% poloxamer to determine the pharmacokinetics over 48 h.

Treatment group	Right flank	Left flank	Rats (*n*)
1	Morphine	Physiological saline	3
2	Morphine	25% Poloxamer	4
3	Morphine with 25% poloxamer	Physiological saline	3
4	Morphine with 25% poloxamer	25% Poloxamer	4

*As a control, 25% poloxamer was injected into eight rats. To randomize the injection sites while maintaining similar physiology, the right and left flanks of each rat were administered either saline for injection, morphine, 25% poloxamer, or morphine with 25% poloxamer as a dosing vehicle for evaluation of tissue irritation of the injection site. Based on the localized subcutaneous injections, there were no anticipated or observed interactions between the morphine and the vehicle controls*.

Blood samples were collected in ethylenediaminetetraacetic acid coated micro tubes. The plasma was separated from the blood and stored at −80°C until analysis with high-performance liquid chromatography (HPLC) coupled to a triple quadrupole mass spectrometer at the Metabolite Profiling Facility, Purdue University Bindley Bioscience Center.

#### Plasma Morphine Concentration Assay

Plasma samples for morphine were prepared for HPLC and mass spectrometry (LC/MS) analysis by doing solid phase extraction first. Supelco Discovery DSC-18 cartridges with 1 ml volume were used for extraction. The individual cartridge was mounted on 10 ml centrifuge tube so that the fluid flushed through the cartridge will be collected in the centrifuge tube. The cartridge was preconditioned by flushing with 3 ml of 100% methanol HPLC grade. Then, 3 ml of double deionized water was used. Before the plasma sample was flushed through the cartridge, 100 µl of the plasma sample was added to 100 µl of D-3 morphine as an internal standard and 1 ml of sodium tetraborate (borax) as buffer. At this point, a new 10 ml centrifuge tube was placed for collection. Two milliliters of methanol were flushed through the cartridge for elution. The collected fluid was transferred to 1.5 ml Eppendorf tube. An Eppendorf vacufuge was used to evaporate the eluate to dryness, which takes approximately 6 h. The mobile phase was later added and stirred well so the residue will be mixed well. The mobile phase used was double deionized water and methanol with a ratio of 3:1.

The plasma morphine concentration was performed on Agilent 1200 series HPLC coupled to Agilent 6460 triple quadrupole mass spectrometer (Agilent Technologies, Santa Clara, CA, USA). The column used with the system for chromatography was Zorbax SB-Phenyl measuring 4.6 mm × 150 mm, 5 µm. The solvents used for mobile phase were 0.1% formic acid in water (solvent A) and 0.1% formic acid in acetonitrile (solvent B). Column flow was set at 0.8 ml/min, and the injection volume was 10 µl. Compound retention time for morphine and D-3 morphine was between 4.0 and 4.3 min. The mass transitions for morphine was 286.1–229.1 and 289.1–229.1 for D-3 morphine. Quantitation standard solutions prepared were from 10 to 1,000 ng/ml. Standard curves had a correlation coefficient of *r*^2^ > 0.99. Sample linearity existed across the range and did not fall outside the upper or lower limits of quantitation.

#### Pharmacokinetic Analysis

The pharmacokinetic parameters were analyzed with a non-compartmental analysis using PKSolver 2.0 for Excel 2010 (Microsoft, Mountain View, CA, USA) as described by Zhang et al. (14). Plasma concentrations of morphine were plotted over time for the M treatment groups (see groups 1 and 2 in Table 1) and compared with PM treatment groups (groups 3 and 4 in Table 1). Relevant pharmacokinetic parameters investigated were the elimination half-life (*t*_1/2_), maximum plasma concentrations (*C*_max_), time to achieve *C*_max_ (*t*_max_), area under the plasma concentration time curve (AUC0 → 48 h), and apparent total body clearance (CL). Absolute bioavailability could not be calculated because an intravenous dose was not performed in this study. Therefore, the CL listed is as per fraction absorbed.

### Histopathological Evaluations of the Injection Sites

At 72 h after treatment, rats were euthanized with carbon dioxide. The skin and tissues of the injection site of the 1 cm × 1 cm demarcation areas were harvested and fixed in a 10% formalin solution. Fixed tissues were embedded in paraffin, sectioned at 6 µm and stained with hematoxylin and eosin. A board-certified pathologist, blinded to the treatment groups performed histological evaluations. Surrounding cell morphology, signs of inflammation and pathological changes were assessed. A summary of the tissue inflammatory scores were used (15). A score of 0 indicated that inflammation was not present, a score of 1 was for a mild degree of inflammation, a score of 2 was for a moderate degree of inflammation, a score of 3 represented a severe degree, and a score of 4 was utilized when the presence of an abscess or other foreign body reaction was observed.

### Statistical Analysis

Statistical analysis was performed using SPSS 23.0 (SPSS Inc., Chicago, IL, USA) with statistical significance set at *p* < 0.05. The Shapiro–Wilk test for normality was performed on all the pharmacokinetic variables. The pharmacokinetic variables were compared between PM treatment and morphine control group. Variables that were normally distributed were analyzed with Student’s *t*-test, otherwise the Mann–Whitney *U* test was performed when the data were not normally distributed. Kruskal–Wallis statistical test was run to compare inflammatory scores for saline, M, poloxamer, and PM treatments.

## Results

### Pharmacokinetic Analysis

**Figure 1:**
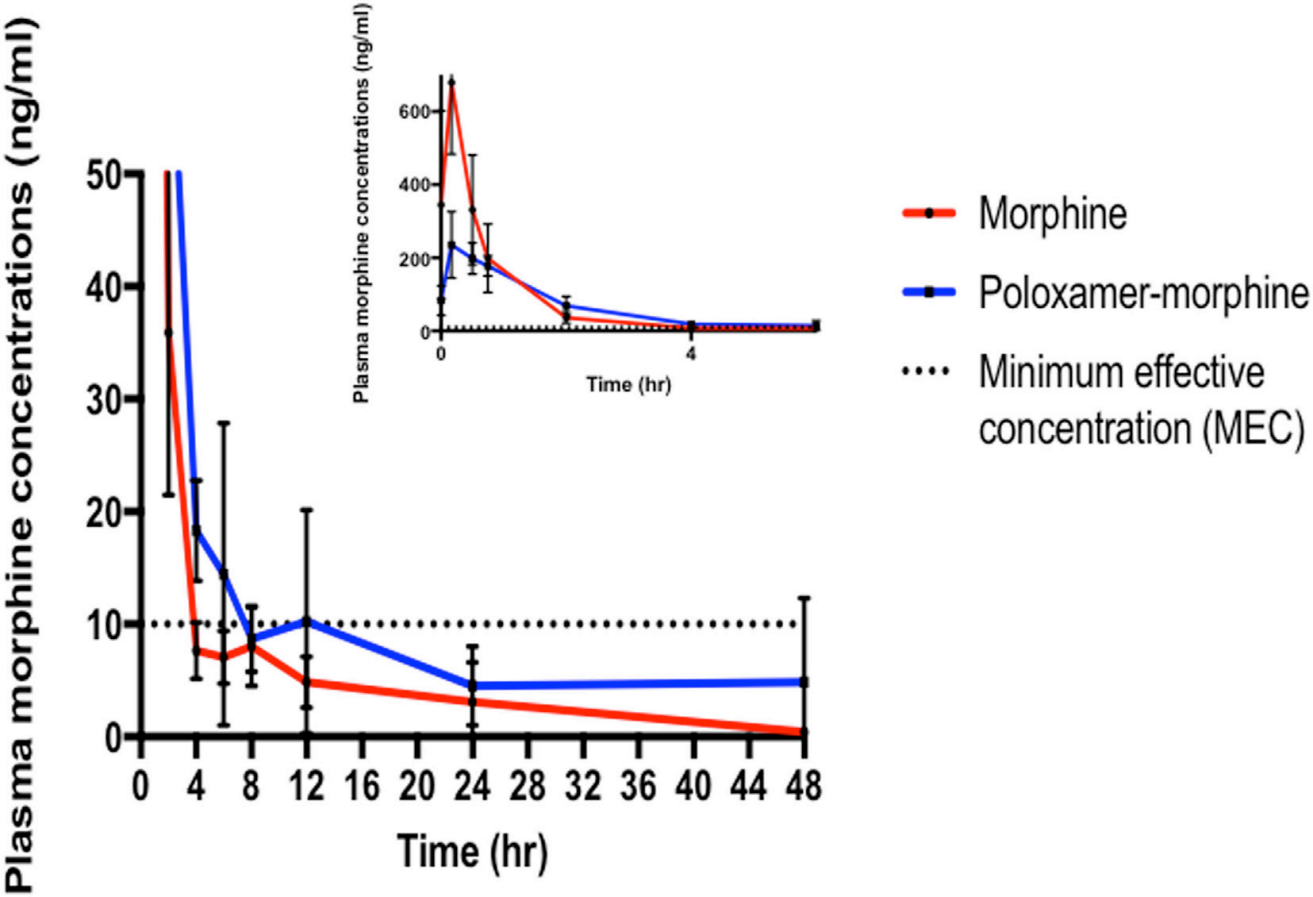
Morphine plasma concentrations (mean ± SD) over time from poloxamer–morphine (in blue) and morphine alone (in red) treated rats from 0 to 48 h after injection; inset figure showing the first 4 h. Please note that there was one outlier in the plasma concentration curve that led to the apparent increase at 12 h, otherwise there would be a more uniform elimination profile.

The summary of the pharmacokinetic results is presented in Table 2. The mean ± SD of morphine plasma concentration over time for poloxamer-morphine and morphine treated rats were presented in Table 3 and can be visualized in Figure 1. From the results, *t*_1/2_ was significantly (*p* = 0.016) longer in the PM group than the M group. The mean *C*_max_ was significantly higher (*p* < 0.001) in the morphine group (716.77 ± 153.65) than with the PM group (242.81 ± 85.53) ng/ml. There was no significant difference between treatment groups in *t*_max_, AUC_0→48h_, and CL. It should be noted that the plasma levels in the PM group were significantly higher at 48 h, thus it is not clear if we would have observed differences had we collected more time points. It also suggests a sustained release effect was observed.

**Table 2:** Morphine plasma concentration (mean ± SD) over time for poloxamer–morphine (PM)- and morphine-treated group.

Time (h)	PM (mean ± SD)	Morphine (mean ± SD)
0	83.38 ± 36.04	344.68 ± 257.53
0.167	235.43 ± 82.77	679.57 ± 198.49
0.5	198.29 ± 39.15	331.04 ± 150.12
0.75	177.75 ± 24.88	198.51 ± 93.58
2	68.60 ± 23.59	35.90 ± 14.41
4	18.32 ± 4.05	7.64 ± 2.50
6	14.45 ± 12.25	7.06 ± 2.35
8	8.68 ± 2.68	8.04 ± 3.51
12	10.23 ± 9.06	4.87 ± 2.25
24	4.51 ± 3.04	3.09 ± 3.52
48	4.85 ± 6.85	0.43 ± 0.40

**Table 3:** Calculated pharmacokinetic parameters following subcutaneous administration of poloxamer–morphine (PM) (2.6 mg/kg) and morphine (1.875 mg/kg).

Parameters	PM (mean ± SD)	Morphine (mean ± SD)	*p*-Value
*C*_max_ (ng/ml)	242.81 (±85.53)	716.77 (±153.65)	<0.001**
*t*_max_ (min)	13.38 (±8.16)	8.34 (±4.08)	0.176
AUC_0–48h_ (ng h/ml)	557.808 (±247.917)	541.410 (±186.298)	0.8995
*t*_1/2_ (h)	5.53 (±7.18)	0.94 (±0.78)	0.016*
CL (L/h/kg)	1.2 (±1.0)	1.0 (±1.0)	

*All of the calculations were dose normalized to control for the variation. In addition, the samples fell within the limits of quantitation by the LC–MS/MS*.

*Clearance (CL) is calculated per fraction of the dose absorbed %F (bioavailability)*.

*p < 0.016; **p < 0.001.

### Histopathology Evaluations

**Figure 2:**
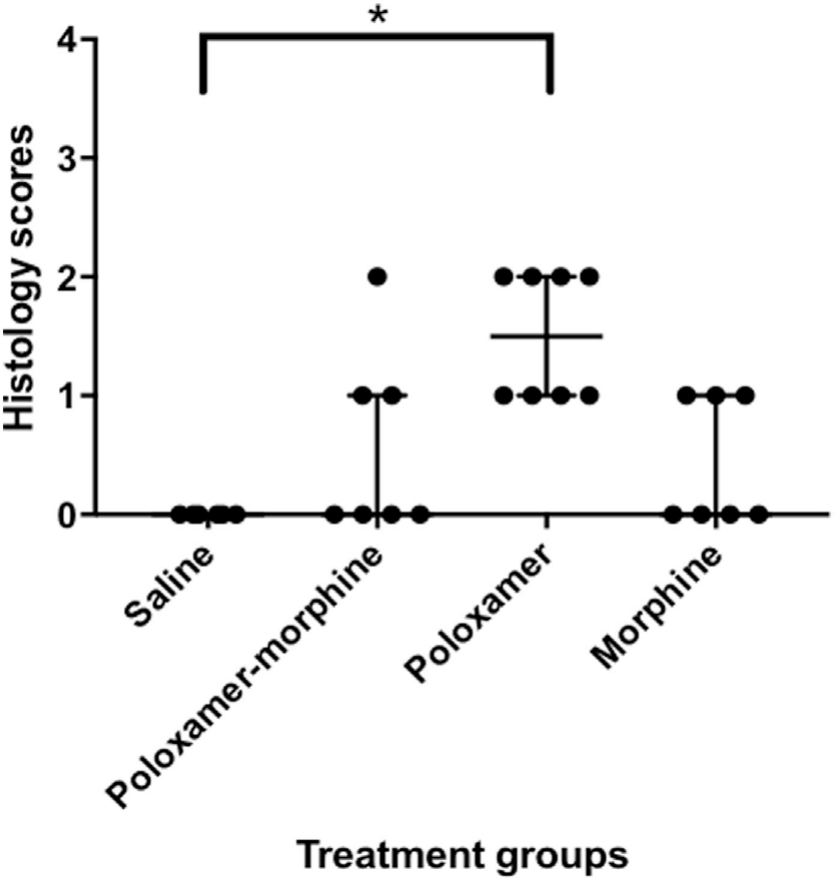
Dot plot with median ± interquartile range (IQR) of histology scores for all treatments. IQR is the difference between the third quartile, Q_3_ and the first quartile, Q_1_. Please note that the poloxamer alone appeared to give an apparently even distribution of either a one or a two scoring. The distribution did not correlate with the morphine dosage form administered in the opposing flank.

**Figure 3:**
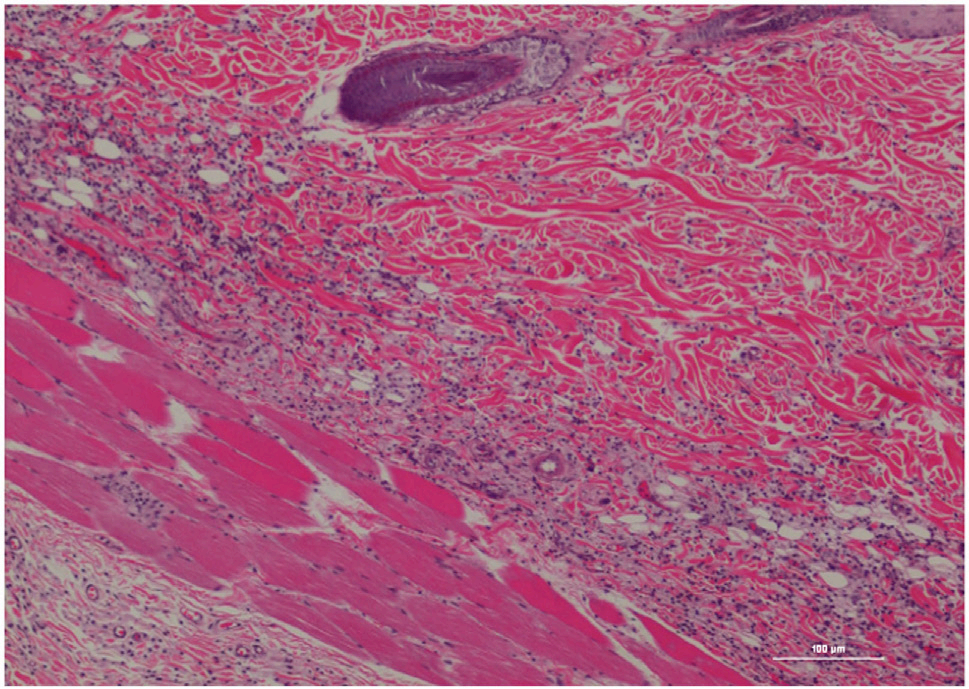
Rat skin biopsy of injection with poloxamer. There was moderate mixed inflammation in deep dermis and panniculus with presence of lymphocyte, neutrophils, macrophage, and scattered mast cells. Hematoxylin and eosin stain. Photomicrograph was taken at 10×. Histology score = 2.

**Figure 4:**
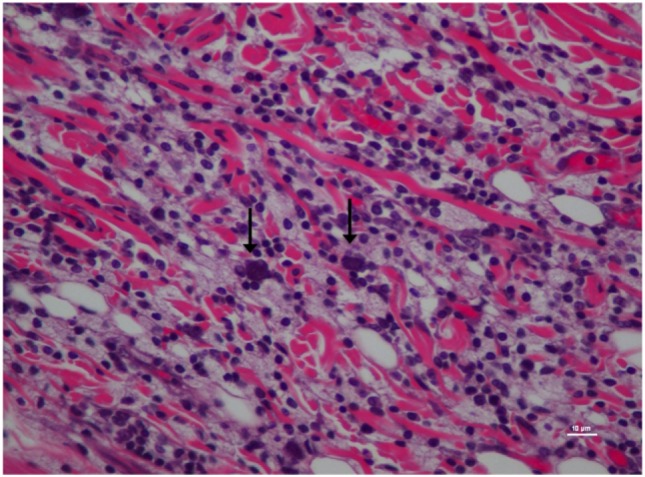
Higher magnification of Figure 3; rat skin biopsy of injection with poloxamer. Majority of the white blood cells population was macrophages with scattered mast cells (vertical arrows). Hematoxylin and eosin stain. Photomicrograph was taken at 40×. Histology score = 2.

The inflammatory scores were similar among morphine, poloxamer, and poloxamer with morphine groups. However, all three groups had significantly higher inflammatory score than the saline control group (see Figure 2). The highest median inflammatory score was from poloxamer injection with a median of 1.5, whereas the other groups all had a median score of 0. All tissue specimens from poloxamer injections (see Figures 3 and 4) showed mild to moderate inflammation perivascularly and in the deep dermis and musculature. In addition, four out of eight poloxamer specimens revealed mild to moderate edema in the subcuticular layer.

**Figure 5:**
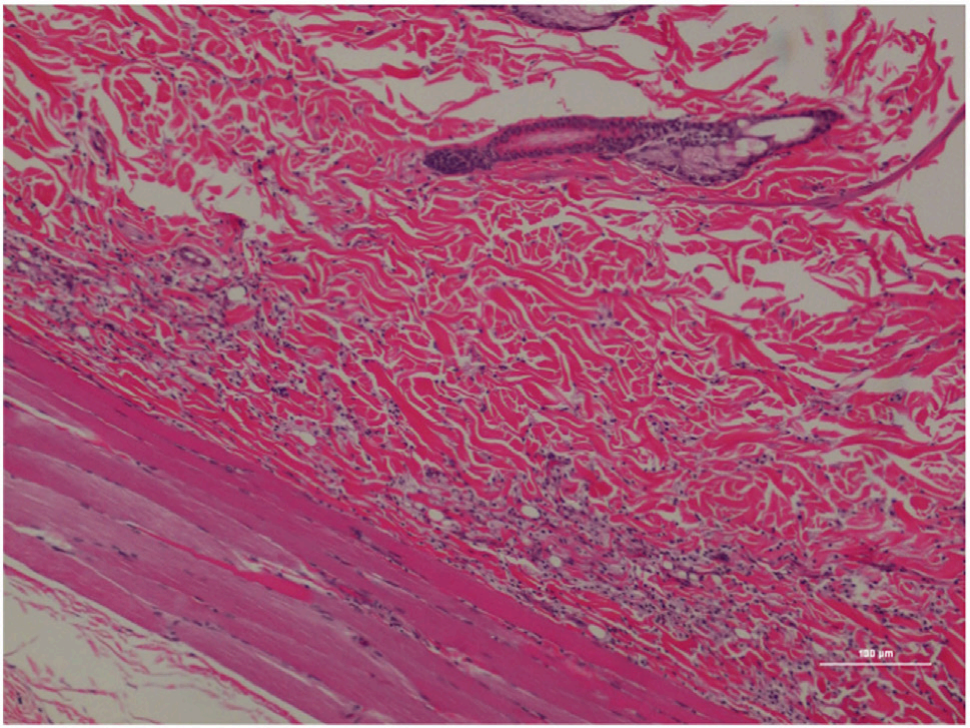
Rat skin biopsy of injection with poloxamer–morphine. There was mild to moderate inflammation in deep dermis and musculature with presence of lymphocyte, plasma cells, and mast cells. Hematoxylin and eosin stain. Photomicrograph was taken at 10×. Histology score = 1.

**Figure 6:**
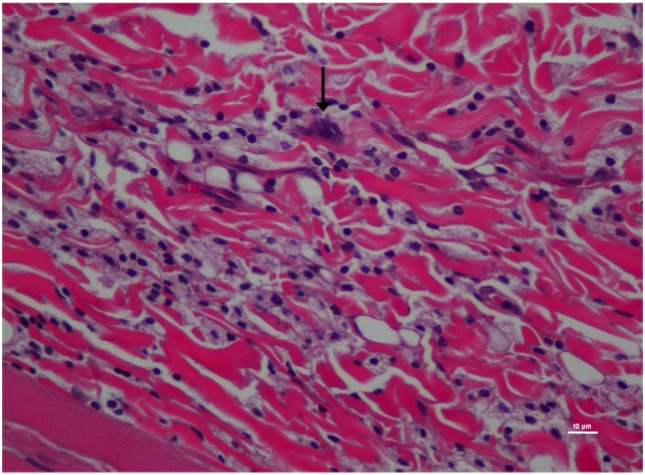
Higher magnification of Figure 5; rat skin biopsy of injection with poloxamer–morphine. Vertical arrow pointing to a mast cell identified by the dense basophillic/blue granules in the cytoplasm. Hematoxylin and eosin stain. Photomicrograph was taken at 40×. Histology score = 1.

**Figure 7:**
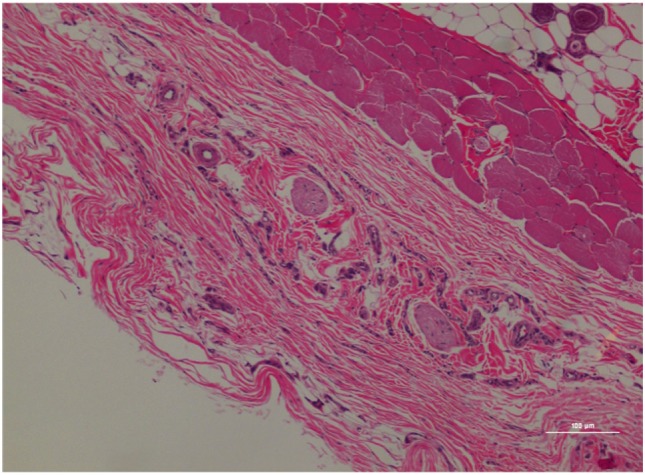
Rat skin biopsy of injection with morphine. There was mild lymphoplasmacytic and mastocytic perivascular inflammation. Hematoxylin and eosin stain. Photomicrograph was taken at 10×. Histology score = 1.

**Figure 8:**
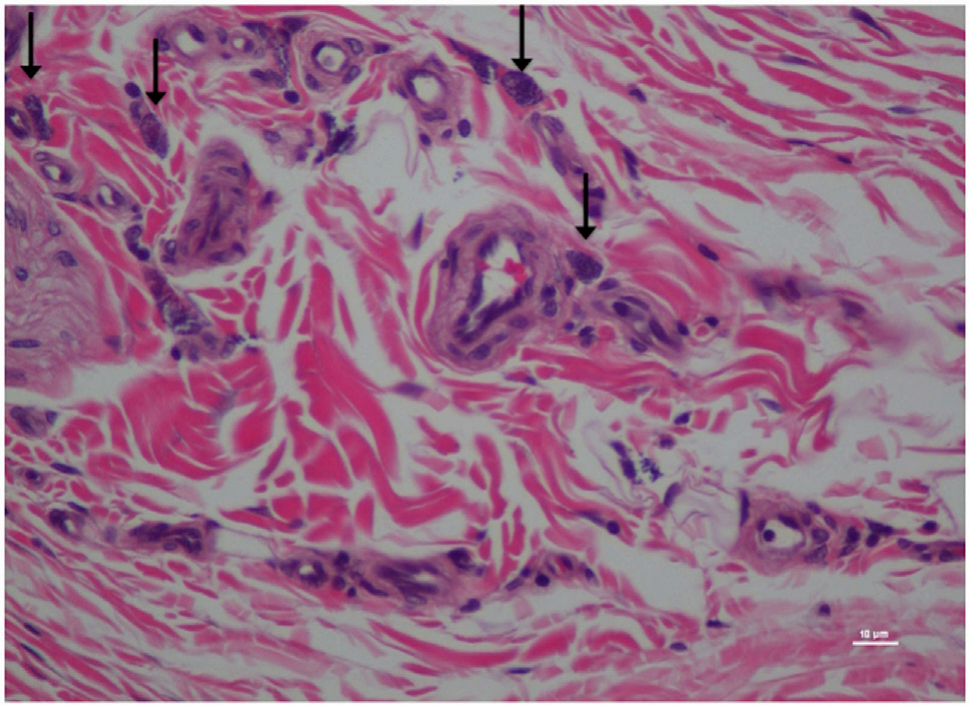
Higher magnification of Figure 7; rat skin biopsy of injection with morphine. Vertical arrow pointing to mast cells identified. Hematoxylin and eosin stain. Photomicrograph was taken at 40×. Histology score = 1.

**Figure 9:**
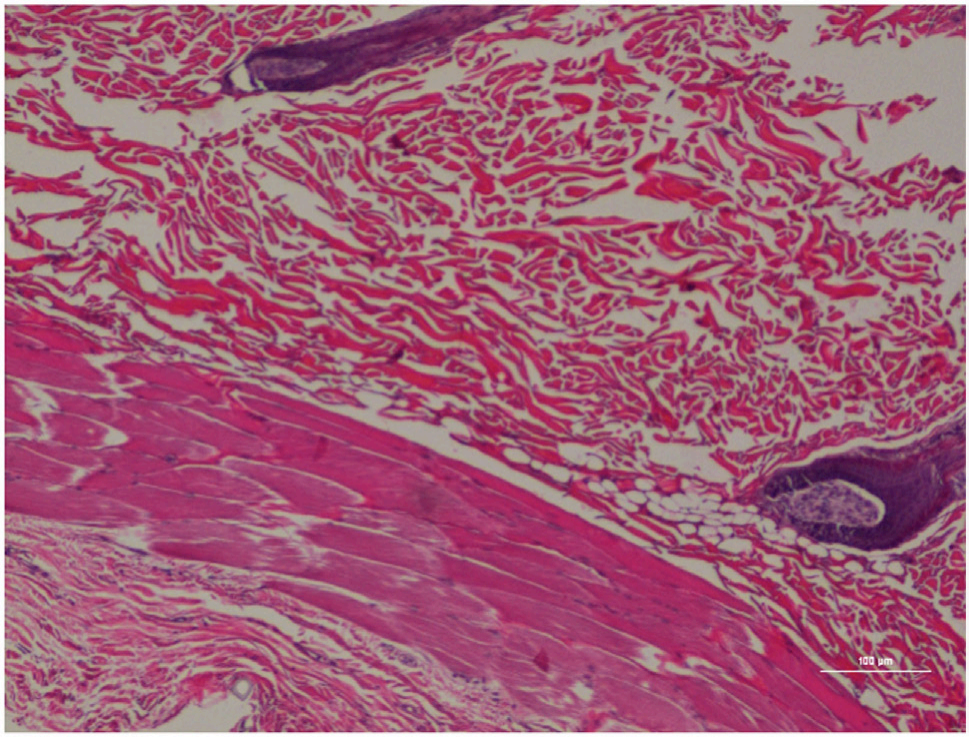
Rat skin biopsy of injection with saline as control. Hematoxylin and eosin stain. Photomicrograph was taken at 10×. Histology score = 0.

The occurrence of inflammation in the deep dermis and musculature was low in the PM specimens (see Figures 5 and 6), where only three out of seven specimens possessed mild to moderate perivascular inflammation and in the accompanying deep dermis and musculature. Similar to poloxamer specimens, none of the PM specimens had a severe inflammation reaction or abscess upon inspection. Specimens from morphine injection (see Figures 7 and 8) revealed perivascular inflammation similar to what was observed with the poloxamer treatment with the presence of lymphocytes, plasma cells, neutrophils, mast cells, and macrophages. Saline injection only showed scant inflammation (see Figure 9).

## Discussion

To the best of our knowledge, this was the first study on the use of poloxamer as a carrier for slowly release of morphine in a rat model. The *t*_1/2_ of morphine in this study was 0.94 ± 0.78 h, which was similar to that reported for dogs and cats (16), but twofold of that reported in mice [0.45 h (17)] and half of the rate in humans [2.1 h (18)]. The observed variability effect of opioid PK across species is well documented (19).

The *C*_max_ of the M-treated rats was approximately three times higher than that of PM-treated rats. This result was not surprising because poloxamer was supposed to bind the morphine in its hydrogel structure and gradually provide a slow release, instead of releasing all of the dose as a bolus upon injected into the rat SC tissues (3).

Our results found that *t*_max_ was similar between M and PM groups, in which both occurred within 15 min of injection, potentially indicating that poloxamer was able to quickly release morphine from its hydroxylated gel pores into the tissues and subsequently absorbed into the rat’s blood circulation. This result was similar to our *in vitro* finding where the PM biocomplex quickly released the morphine into the culture media (12). However, we cannot rule out that the observed *t*_max_ was not due to other factors including a release of untrapped morphine in the polaxamer hydrogel, a prolonged hydrogel formation period that enabled to escape of some residual morphine, or that the poloxamer concentration was not sufficient to encapsulate all of the morphine in the formed hydrogel. Additional research on optimizing the formulation parameters is ongoing.

The plasma morphine concentration patterns after the drug injection were similar between M and PM treatment groups, characterized by having a rapid increasing and decreasing levels in the first 45 min after drug administration. By 45 min after the drug administration, the plasma morphine concentrations in both treatment groups had rapidly decreased from their *C*_max_ and reached similar plasma concentrations at this time (Figure 1). There appeared to be higher morphine plasma concentrations in the PM group than those administered M alone at 2 h (Table 2). This was a distinct inflection in the plasma concentrations versus time profiles that was maintained throughout the remaining respective time points between the M and PM treatments. The inflection and resulting higher morphine concentrations appear to indicate that the PM mixture continues to elute morphine whereas the M alone injection began a rapid decline and elimination dominated. Despite the significant differences of the respective *C*_max_ values, the AUC0 → 48 h was nearly identical between M and PM treatments. This most likely signifies that poloxamer was capable of sustaining the release of morphine from its hydrogel structure into rat’s blood circulation over a prolonged period, as expected. The therapeutic significance was not assessed in this proof of concept study, although it may be implied that analgesia could be attenuated for a prolonged period utilizing the hydrogel forming poloxamer drug delivery system under optimized conditions.

Among all the histopathological evaluations, poloxamer alone injection showed the highest median inflammatory score at 1.5. However, no severe inflammatory reaction or abscess was found in any of the treatment groups, with the highest score was at 2. The gross pathological examination of the harvested tissue specimens appeared to reveal that no residual poloxamer material was observed in the SC tissues, consistent with its proposed biodegradable properties that make it favored as a drug vehicle of choice in the field. When the skin specimens were harvested for histological evaluation, all tissues with the exception of two showed no obvious gross abnormality. As for the exceptions, one specimen had a hematoma of the subcuticular tissue, and another specimen from a different rat at the point of a needle entry site. The observed hematoma was likely caused by a needle injury during injection.

In our study, we observed that almost half of our morphine administered specimens showed mild perivascular inflammation, without a pattern relating to the absence or presence of polaxamer. In humans, SC infusion of morphine has been associated with local tissue irritation like erythema and swelling (20). Injectable morphine solution is usually kept in acidic pH due to the compound being more stable in the acidic environment and the increased potential of oxidation under more alkaline pH levels (21). The slight acidity could be one of the potential causes for local tissue injury by morphine injection. Based on this finding, clinicians may want to reconsider the potential tissue injury associated with repeated morphine injections when given as SC or intramuscular injections or investigate reducing repeated injections by using sustained release delivery vehicles like we have described here.

It is also interesting that the mixture of morphine with poloxamer injection did not exacerbate the host reaction to cause a more severe inflammatory reaction, despite the fact that the poloxamer alone did cause more inflammation. One potential explanation may lie in the fact that the PM shows a less severe inflammation compared with poloxamer alone through a dilution and potential local morphine analgesia effect with acute usage. To elucidate the exact cause would warrant further investigation, but it might be interesting to compare the histology score of different concentrations of the mixture to help explain the scenario.

## Conclusion

In this study, we demonstrated that morphine in combination with 25% poloxamer was able to sustain the release of morphine (longer *t*_1/2_) from the resultant hydrogel structure over a longer duration than morphine alone. The *C*_max_ for morphine alone was much higher but the other pharmacokinetic profiles were similar (*t*_max_, AUC0 → 48 h and CL). In addition, the PM did not cause a significant tissue inflammation with an acute SC administration in rats. Further studies are needed in assessing the analgesic efficacy of PM in the clinical setting.

## Ethics Statement

This work was conducted at Purdue University’s AAALAC-accredited facility and was approved by Purdue’s IACUC (1412001174).

## Author Contributions

All persons who meet authorship criteria are listed as authors. The initials for each author are NS, JK, YJ-H, H-YW, MD, GB, and GK. Conception and design of the study; drafting or revising manuscript critically for important intellectual content; and final approval of version to be published: NS, JK, YJ-H, H-YW, MD, and GB. Acquisition of data: NS and YJ-H. Analysis and/or interpretation of data: H-YW and GK.

## Conflict of Interest Statement

The authors declare that the research was conducted in the absence of any commercial or financial relationships that could be construed as a potential conflict of interest.
